# C-855-05. Ethics Meets Economics: A Cost-Aware Framework for Burn Triage in Mass Casualty Incidents

**DOI:** 10.1093/jbcr/irag033.089

**Published:** 2026-04-06

**Authors:** Moon Usman, Emily D Dubina, Nicholas Sheets, Roselle E Crombie

**Affiliations:** Riverside Community Hospital, Riverside, CA; Riverside Community Hospital, Riverside, CA; Riverside Community Hospital, Riverside, CA; Connecticut Burn Center/Yale New Haven Health, Westport, CT

## Abstract

**Introduction:**

Mass burn casualty incidents (MBCIs) overwhelm health systems, forcing crisis standards of care. Ethical allocation must balance equity, duty to care, and resource stewardship. Prior austere-care frameworks lacked integration of modern survival predictors, resource modeling, and financial impact. This study develops a hybrid triage framework combining the Outcomes-to-Resources Ratio (ORR), updated survival data, and cost-of-care estimates, aligned with the updated 2024 Western Regional Burn Disaster Consortium (WRBDC) operations plan.

**Methods:**

A structured synthesis was performed. First, guidelines from the American Burn Association and the 2024 Western Regional Burn Disaster Consortium (WRBDC) operations plans were reviewed to define constraints including the 72-hour delay before federal resource arrival.(1,2,7) Survival estimates were extracted from the ABA Burn Injury Summary Report (2024) and recent predictors of mortality (TBSA, respiratory SOFA).(1,8) Additional resource utilization was modeled using the ORR framework, linking survival probability to ICU-day demand.(6) A cost analysis was then applied utilizing Society of Critical Care Medicine benchmarks ($4500–6000/day) and adjusted upward 1.5–2x for burn ICU complexity.(5) Finally, quantitative results were mapped to ethical principles of fairness, proportionality, and transparency to define triage categories.

**Results:**

Survival varied significantly by age and burn size. Patients under 40 years with ≤20% TBSA had >95% survival, while patients over 70 years with >50% TBSA fell below 50%.(1,8) ORR modeling showed that an 18-year-old with 50% TBSA required ~20 ICU days with >80% survival, while a 75-year-old with the same injury required >30 ICU days with <20% survival.(6) Cost analysis indicated the younger patient’s ICU stay corresponded to ~$180 k–240 k per survivor, whereas the older patients exceeded $270 k–360 k, translating to >$1 M per life saved.(5) Ethical mapping supported three triage categories: High-Priority (≥50% survival with feasible resources and cost), Intermediate (uncertain benefit; daily reassessment), and Expectant (< 15% survival despite maximal support and disproportionate cost).

**Conclusions:**

This hybrid ethical–quantitative framework integrates survival, resource demand, and cost-of-care into a reproducible triage model. It provides a transparent, equitable, and economically informed approach to allocation during MBCIs. Additionally, it can guide planners with personnel directives and aid in predicting future needs in austere environments.

**Applicability of Research to Practice:**

The model can be incorporated into regional disaster planning, tabletop exercises, and provider training. By linking outcomes, ICU utilization, and cost, it supports fair and defensible decisions under crisis standards and maximizes lives saved per resource expended.

**Funding for the study:**

N/A.

